# Granulosa Cell‐Layer Stiffening Prevents Escape of Mural Granulosa Cells from the Post‐Ovulatory Follicle

**DOI:** 10.1002/advs.202403640

**Published:** 2024-07-01

**Authors:** Xiaodong Wang, Jianning Liao, Hongru Shi, Yongheng Zhao, Wenkai Ke, Hao Wu, Guoshi Liu, Xiang Li, Changjiu He

**Affiliations:** ^1^ National Center for International Research on Animal Genetics, Breeding and Reproduction / Key Laboratory of Agricultural Animal Genetics, Breeding and Reproduction of Ministry of Education, College of Animal Science and Technology Huazhong Agricultural University Wuhan 430070 P. R. China; ^2^ National Engineering and Technology Research Center for Livestock Wuhan 832003 P. R. China; ^3^ Hubei Provincial Center of Technolgy Innovation for Domestic Animal Breeding Wuhan 100193 P. R. China; ^4^ College of Animal Science and Technology Shihezi University Shihezi 832003 P. R. China; ^5^ Key Laboratory of Animal Genetics and Breeding of the Ministry of Agriculture, College of Animal Science and Technology China Agricultural University Beijing 100193 P. R. China

**Keywords:** focal adhesion, follicle, granulosa cell, luteinization, ovulation

## Abstract

Ovulation is vital for successful reproduction. Following ovulation, cumulus cells and oocyte are released, while mural granulosa cells (mGCs) remain sequestered within the post‐ovulatory follicle to form the corpus luteum. However, the mechanism underlying the confinement of mGCs has been a longstanding mystery. Here, in vitro and in vivo evidence is provided demonstrating that the stiffening of mGC‐layer serves as an evolutionarily conserved mechanism that prevents mGCs from escaping the post‐ovulatory follicles. The results from spatial transcriptome analysis and experiments reveal that focal adhesion assembly, triggered by the LH (hCG)‐cAMP‐PKA‐CREB signaling cascade, is necessary for mGC‐layer stiffening. Disrupting focal adhesion assembly through RNA interference results in stiffening failure, mGC escape, and the subsequent development of an abnormal corpus luteum characterized by decreased cell density or cavities. These findings introduce a novel concept of “mGC‐layer stiffening”, shedding light on the mechanism that prevents mGC escape from the post‐ovulatory follicle.

## Graphical abstract

Stimulation by ovulatory signals LH or hCG augments the cAMP‐PKA‐CREB cascade in the mural granulosa cells (mGCs). This cascade initiates focal adhesion assembly within the mGC‐layer, which substantially fortifies the adhesive interactions between the mGCs and the extracellular matrix (ECM). Consequently, the mGC‐layer becomes more rigid. The augmented rigidity diminishes the layer's flexibility, preventing the escape of mGCs through the ruptured follicle.

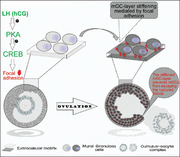

## 1 Introduction

Ovulation, a pivotal event in female reproduction, marks both the culmination of oogenesis and the commencement of luteinization. This event is triggered by ovulatory signals, specifically the luteinizing hormone (LH) surge or human chorionic gonadotropin (hCG). The pre‐ovulatory follicle, which has the potential to ovulate, is a sophisticated structure mainly composed of mural granulosa cells (mGCs), cumulus cells, an oocyte, and theca cells. Each follicular cell type plays an indispensable role in coordinating the programmed ovulation process and luteinization.^[^ ^1^, ^2^ ^]^ Apart from the aforementioned follicular cells, ovarian stromal cells including immune cells, vascular cells, and perivascular cells also contribute significantly to normal ovulation and luteinization.^[^ ^3^, ^4^ ^]^

Upon receiving an ovulatory signal, different types of follicular cells undergo distinct fates. The oocyte resumes meiosis, extrudes the first polar body, and is released from the ruptured follicle.^[^ ^5^, ^6^ ^]^ Concurrently, the cumulus cell layer undergoes extracellular matrix (ECM) remodeling, expansion, and increasing viscosity,^[^ ^7^ ^]^ along with the production of numerous inflammatory mediators.^[^ ^8^, ^9^ ^]^ Eventually, the cumulus cells accompany the oocyte as it migrates to the ampulla of the fallopian tube. mGCs function to receive and convey the ovulatory signals. Abundant LH receptors on the mGC cytomembrane allow for sensitive recognition of the signals.^[^ ^10^, ^11^ ^]^ Moreover, signaling cascades like the EGF signaling and MAPK3/1 signaling in mGCs play a crucial role in amplifying the ovulatory signal and transmitting it to cumulus cells and oocyte.^[^ ^12^, ^13^ ^]^

Following ovulation, cumulus cells and oocyte are released, while mGCs remain sequestered within the post‐ovulatory follicle to give rise to the corpus luteum, crucial for regulating the estrus cycle and supporting pregnancy. Interest is piqued by the fact that cumulus cells and mGCs have a common progenitor in preantral follicles.^[^ ^14^, ^15^ ^]^ It is puzzling, therefore, to consider why cumulus cells can escape the post‐ovulatory follicle, while mGCs, stemming from the same precursor, lack this capability. Thus far, no theoretical model has been developed to address this question.

Our investigation uncovered that the mGC‐layer experiences a unique process called “mGC‐layer stiffening” upon receiving the ovulatory signals. This stiffening prevents the mGC‐layer from escaping the ruptured follicle. The results from spatial transcriptome analysis and in vitro and in vivo experiments revealed that focal adhesion assembly, triggered by the LH (hCG)‐cAMP‐PKA‐CREB signaling cascade, is necessary for mGC‐layer stiffening. Disrupting focal adhesion assembly through RNA interference resulted in failed mGC‐layer stiffening and subsequent release of mGCs from the post‐ovulatory follicle.

## 2 Results

### 2.1 Ovulatory Signals Triggered the Stiffening of mGC‐Layer and Prevented its Escape from the Punctured Follicle

**Figure 1:**
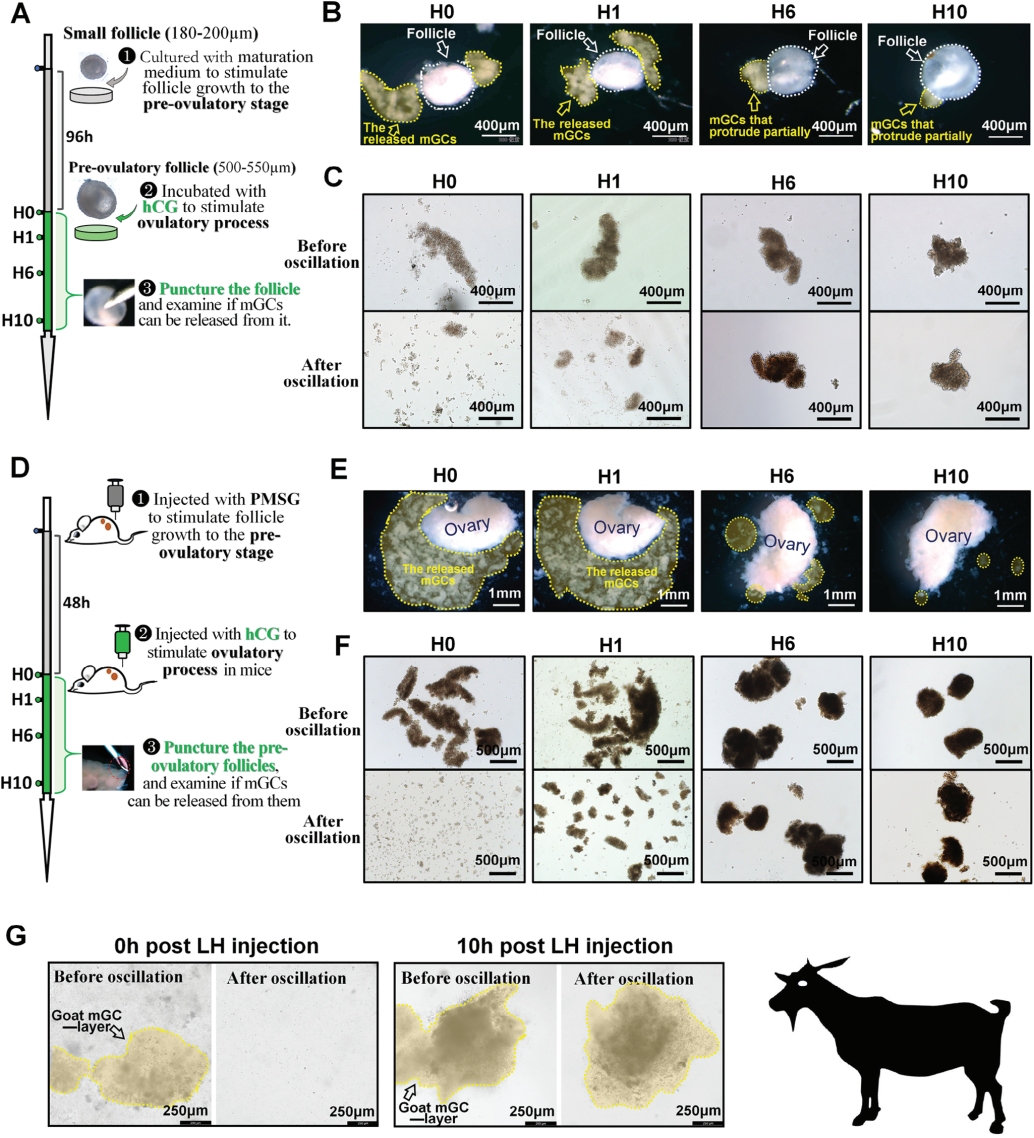
Ovulatory signals triggered the stiffening of mGC‐layer and prevented its escape from the punctured follicle. A) Experimental design for *B* and *C*. B) Effect of hCG addition on the capability of mGC‐layer to escape from punctured follicles. The mGC‐layers are outlined by yellow frames, and the follicles are outlined by white frames. C) Effect of hCG addition on the rigidity of mGC‐layer. Oscillation parameter: 700 rpm, 1 min, and 37 °C. D) Experimental design for *E* and *F*. E) Effect of hCG injection on the capability of mGC‐layer to escape from punctured ovaries. The mGC‐layers are outlined by yellow frames. F) Effect of hCG injection on the rigidity of mGC‐layer. Oscillation parameter: 700 rpm, 1 min, and 37 °C. G) Effect of LH injection on the rigidity of goat mGC‐layer. Oscillation parameter: 700 rpm, 1 min, and 37 °C. The mGC‐layers are outlined by yellow frames. B, C, E, F were repeated independently five times, and G was repeated two times, yielding consistent results.

To facilitate real‐time monitoring and studying of the ovulation process, we developed a mouse follicle culture system capable of supporting ovulation and luteinization while allowing for gene knockdown within the follicle (**Figure** **1**A, Experimental Section). By puncturing the cultured follicles before or after the addition of hCG, we observed distinct outcomes in the release of mGCs. Puncturing before hCG addition (H0) or 1 h after hCG addition (H1) exhibited facilitated release of mGCs, while at 6 (H6) and 10 h (H10) post hCG addition, minimal or no mGCs were observed to be released (Figure 1B; Movie S1, Supporting Information). Importantly, we observed a significant increase in the rigidity of the mGC‐layer following hCG addition. The mGC‐layers exhibited low rigidity at H0 and H1, fragmenting under mechanical oscillation, but demonstrated enhanced rigidity at H6 and H10, enabling them to maintain structural integrity when exposed to the same concussive force (Figure 1C). We termed this phenomenon “mGC‐layer stiffening”.

We further investigated the phenomena of mGC‐layer stiffening in vivo using the superovulation technique (Figure 1D). Consistent with our in vitro findings, hCG injection induced mGC‐layer stiffening and prevented mGC release from the punctured ovary. Briefly, at H0 and H1, the unstiffened mGC‐layer burst out of the punctured ovarian surface (Figure 1E; Movie S2, Supporting Information) and disintegrated upon mechanical oscillation (Figure 1F; Movie S3, Supporting Information), while at H6 and H10, the stiffened mGC‐layer remained trapped in the ovary with only a few mGCs released (Figure 1E; Movie S2, Supporting Information). These stiffened mGC clumps remained intact after mechanical oscillation (Figure 1F; Movie S3, Supporting Information). Our observations led to the hypothesis that the ovulatory signal‐triggered mGC‐layer stiffening prevents mGCs escape from the post‐ovulatory follicle.

Interestingly, we also observed the phenomena of mGC‐layer stiffening in goats. Before LH injection, the mGC‐layer in goats exhibited low rigidity, disintegrating after mechanical oscillation. However, at 10 h post LH injection, the mGC‐layer displayed increased rigidity and remained intact under concussive force (Figure 1G).

### 2.2 Spatial Transcriptome Analysis Suggested that mGC‐Layer Stiffening may Result from the Assembly of Focal Adhesions

**Figure 2:**
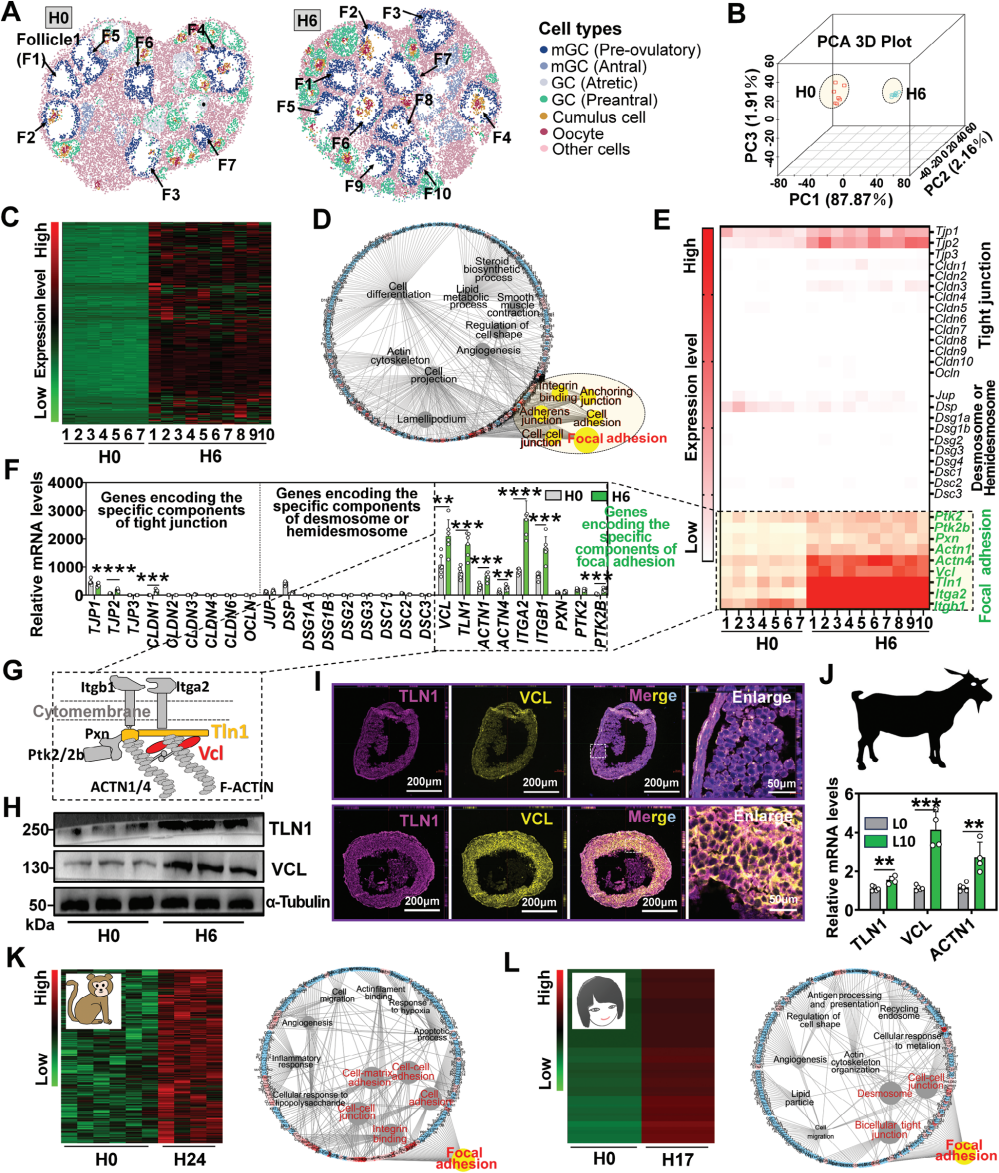
Spatial transcriptome analysis suggested that mGC‐layer stiffening may result from the assembly of focal adhesions. A) Identification of pre‐ovulatory follicles within the ovaries through spatial transcriptome analysis. n = 7 (H0) and 10 (H6) pre‐ovulatory follicles, respectively. The mGCs within the pre‐ovulatory follicles are highlighted in blue. B) PCA analysis of the transcriptome differences in mGCs within pre‐ovulatory follicles. C) Heat map of the up‐regulated genes in mGCs after hCG injection. D) GO analysis of the up‐regulated genes. Biological processes related to cell adhesion are outlined by yellow frames. E) Heat map of genes encoding the components of tight junction, desmosome, hemidesmosome, and focal adhesion. F) qRT‐PCR validation of the expression of genes encoding the components of tight junction, desmosome, hemidesmosome, and focal adhesion. n = 6 mGC samples. G) Schematic representation of the structure of a focal adhesion. H) Western blot assay of the protein contents of VCL and TLN1 after hCG injection. n = 3 mGC samples. Original blots can be viewed in Figure S4A (Supporting Information). I) Immunofluorescence analysis of the localization of VCL and TLN1 in follicle after hCG injection. Nuclei (blue) were stained with DAPI. J) qRT‐PCR analysis of the expression of goat genes encoding the components of focal adhesion after LH injection. L0: 0 h post LH injection, L10: 10 h post LH injection. n = 5 (L0) and 4 (L10) mGC samples. K) Analysis of the transcriptome changes in monkey mGC after hCG injection. *Left*: Heat map of the up‐regulated genes; *right*: GO analysis of the up‐regulated genes. Focal adhesion outlined by yellow. L) Analysis of the transcriptome changes in human mGC after hCG injection. *Left*: Heat map of the up‐regulated genes; *right*: GO analysis of the up‐regulated genes. Focal adhesion outlined by yellow. Statistical significance was determined using two‐tailed unpaired Student's t‐test, values were mean ± SD. ^**^ *p *< 0.01, ^***^ *p *< 0.001, ^****^ *p *< 0.0001. F, H, and I were repeated independently three times, yielding consistent results.

We conducted spatial transcriptomic analysis of the ovaries of H0 and H6 to explore the mechanisms underlying the occurrence of mGC‐layer stiffening. Spatial transcriptome data were acquired from public databases.^[^ ^16^ ^]^ A total of 7 pre‐ovulatory follicles at H0 and 10 at H6 were identified, respectively. From these follicles, we obtained transcriptional dynamics of mGCs following hCG stimulation (**Figure** **2**A). Principal component analysis (PCA) demonstrated significant transcriptional differences among these mGCs (Figure 2B). A total of 5352 differentially expressed genes were identified using the DESeq2 software. Among these genes, 2303 were found to be up‐regulated after hCG injection (Figure 2C). Gene ontology (GO) analysis demonstrated that the up‐regulated genes were mainly enriched in biological processes related to cell adhesion, such as Focal Adhesion, Cell‐cell Junction, Integrin Binding, and Anchoring Junction, in addition to well‐known ovulation‐related processes (Figure 2D). This led us to hypothesize that alterations in cell adhesion contribute to mGC‐layer stiffening.

To investigate the specific type of cell adhesion involved in achieving mGC‐layer stiffening, we examined genes encoding components of tight junction, desmosome, hemidesmosome, and focal adhesion. Analysis of the spatial transcriptomic data showed that only genes encoding components of focal adhesions were highly expressed and upregulated by hCG in mGCs (Figure 2E). To validate the transcriptome data, quantitative real‐time PCR (qRT‐PCR) was conducted, demonstrating consistent gene expression patterns (Figure 2F). Furthermore, western blot assay (Figure 2H) and dual fluorescent staining (Figure 2I) confirmed the significant upregulation of VCL and TLN1, fundamental structural proteins of focal adhesions (Figure 2G), in the mGC‐layer post‐hCG injection. Particularly striking was the strong co‐localization of TLN1 with VCL observed in H6 follicles, in contrast to H0 follicles. Moreover, the co‐localization signals of TLN1 with VCL were dispersed throughout the mGC‐layer, indicating that focal adhesion assembly occurs throughout the mGC‐layer.

Additionally, the qRT‐PCR assay also showed upregulation of *VCL*, *TLN1*, and *ACTN1* in goat mGCs after LH injection (Figure 2J). Analysis of transcriptome data from other species revealed that focal adhesion assembly after the ovulatory signal stimulation is not exclusive to mice but also occurs in monkey and human (Figure 2K,L).^[^ ^17^, ^18^ ^]^ These findings indicate that ovulatory signal‐induced focal adhesion assembly is a conserved event across species. We hypothesized that ovulatory signals induce focal adhesion assembly, leading to the occurrence of mGC‐layer stiffening.

### 2.3 Disruption of Focal Adhesion Assembly Led to a Failure in the Stiffening of mGC‐Layer and an Escape of mGCs from the Manually Punctured Follicle

**Figure 3:**
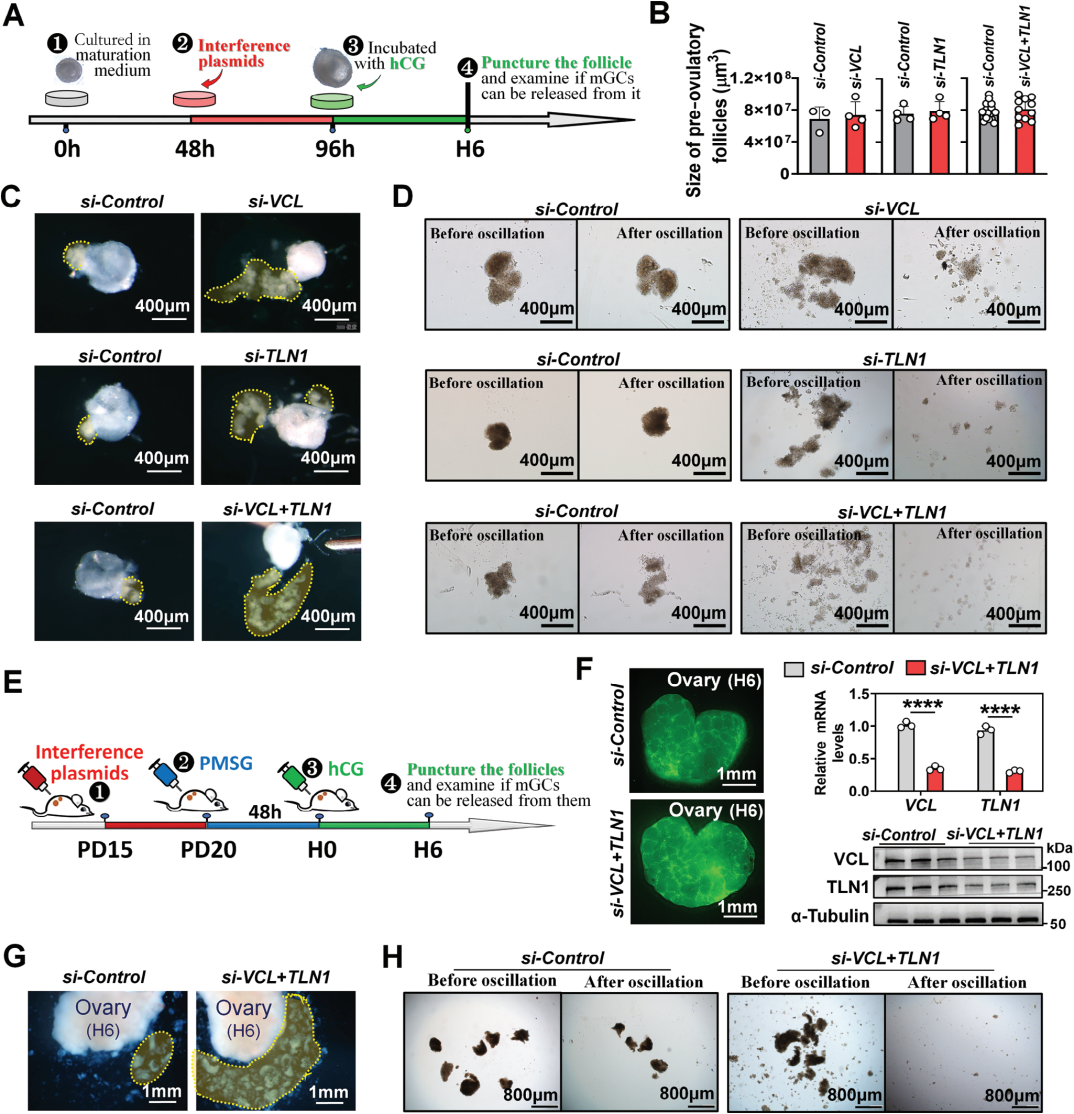
Disruption of focal adhesion assembly led to a failure in the stiffening of mGC‐layer and an escape of mGCs from the punctured follicle. A) Schematic representation of the knockdown of *VCL* and *TLN1* in cultured follicles. B) Effect of *VCL* and *TLN1* knockdown on follicle growth, n = 3 (*si‐Control*), 4 (*si‐VCL*); 4 (*si‐Control*), 4 (*si‐TLN1*); 18 (*si‐Control*), 11 (*si‐VCL+TLN1*). The scrambled shRNA was used as *si‐Control* in this study. C) Effect of *VCL* and *TLN1* knockdown on the capability of mGC‐layer to escape from the punctured follicles. The mGC‐layers are outlined by yellow frames. D) Effect of *VCL* and *TLN1* knockdown on the rigidity of mGC‐layer. Oscillation parameters: 700 rpm, 1 min, and 37 °C. E) Schematic representation of the knockdown of *VCL* and *TLN1* in ovaries. F) Efficiency analysis of *VCL*+*TLN1* interference using qRT‐PCR and western blot, n = 3 ovaries, collected from 3 mice. Green fluorescence indicates successful transcription of interfering plasmids in ovaries. Original blots can be viewed in Figure S4B (Supporting Information). G) Effect of *VCL*+*TLN1* knockdown on the capability of mGC‐layer to escape from punctured ovaries. mGC‐layers outlined by yellow frames. H) Effect of *VCL*+*TLN1* knockdown on the rigidity of mGC‐layer. Oscillation parameter: 700 rpm, 1 min, and 37 °C. Statistical significance was determined using two‐tailed unpaired Student's t test, with values presented as mean ± SD. ^****^ *p *< 0.0001. C, D were repeated independently five times, and G, H were repeated two times, yielding consistent results.

In order to validate our hypothesis concerning the relationship between focal adhesion assembly and mGC‐layer stiffening, we employed lentivirus‐mediated RNA interference to disrupt the assembly of focal adhesions. Focal adhesion is composed of various components, including talin (TLN1), vinculin (VCL), paxillin, α‐actinin, and focal adhesion kinase (FAK), and other proteins.^[^ ^19^ ^]^ TLN1 and VCL, in particular, are indispensable structural proteins in focal adhesion. Absence either of them leads to a notable impairment of focal adhesion.^[^ ^20^, ^21^, ^22^ ^]^ Given this, we opted to knock down *TLN1* and *VCL* to disrupt the assembly of focal adhesions (**Figure** **3**A). The result indicated that transfecting plasmids 48 h prior to the addition of ovulation‐inducing medium can effectively decrease the mRNA (Figure S1A–C, Supporting Information) and proteic (Figure S1D, Supporting Information) levels of VCL and TLN1 at H6, and weak the co‐localization signal of VCL and TLN1 in mGC‐layer (Figure S1E, Supporting Information), without exerting adverse impact on the growth of follicles to the pre‐ovulatory stage (Figure 3B). However, the knockdown of *VCL* and *TLN1*, either individually or in combination, led to the unstable retention of mGCs within the follicles at H6. Upon puncturing the follicles, mGCs in the *si‐VCL* (Movie S4, Supporting Information), *si‐TLN1* (Movie S5, Supporting Information), and *si‐VCL+TLN1* (Movie S6, Supporting Information) groups easily burst out, while those in the control groups struggled to escape (Figure 3C). Furthermore, we discovered that the disruption of focal adhesion assembly hindered the stiffening of the mGC‐layer, as the mGC‐layers in the *si‐VCL*, si‐*TLN1*, and *si*‐*VCL+TLN1* groups showed lower rigidity and disintegrated upon mechanical oscillation compared to the control groups (Figure 3D).

To further validate our findings, we performed injections of lentiviral particles beneath the ovarian bursa to specifically knock down the mRNA and proteic levels of VCL and TLN1 in the ovaries (Figure 3E,F). Consistent with our in vitro observations, simultaneous knockdown of *VCL* and *TLN1* facilitated the release of mGC‐layers from the ovaries at H6 (Figure 3G). Notably, the mGC‐layer in the *si‐VCL+TLN1* group exhibited reduced rigidity, leading to its disintegration upon mechanical oscillation compared to the control group (Figure 3H). These findings provide compelling evidence supporting the pivotal role of focal adhesion assembly, induced by the ovulatory signals, in the stiffening of the mGC‐layer and its subsequent inability to escape from manually punctured follicles.

### 2.4 Disruption of Focal Adhesion Assembly Resulted in the Release of mGCs from the Post‐Ovulatory Follicle and a Reduction in the Quantity of Luteal Cells

**Figure 4:**
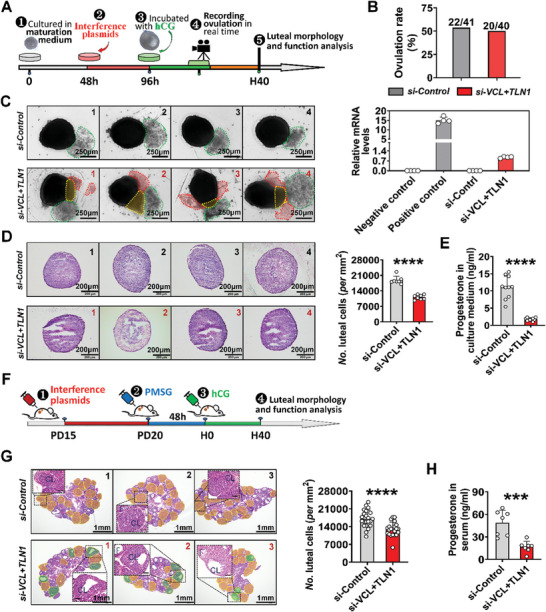
Disruption of focal adhesion assembly resulted in the release of mGCs from the post‐ovulatory follicle and a reduction in the quantity of luteal cells. A) Schematic representation for real‐time recording of ovulation process after *VCL+TLN1* knockdown. B) Effect of *VCL+TLN1* knockdown on the ovulation rate. C) Knocking down *VCL+TLN1* results in the spontaneous release of mGCs from the post‐ovulatory follicle. *Left*: Representative photographs of the post‐ovulatory follicles. The free mGCs released from the rupture site are outlined by red frames; the mGC clumps protruded from the rupture site are outlined by yellow frames; the released COCs are covered by green frames. *Right*: identity verification of the released mGCs through qRT‐PCR. n = 4 cellular samples. *Lhcgr* was chosen as the marker gene for mGC. Purified mGCs and cumulus cells were used as positive and negative controls, respectively. D) Effect of *VCL+TLN1* knockdown on the morphology and function of the in vitro corpus luteum. *Left*: representative photographs of luteal sections in each group. *Right*: statistics of the density of luteal cells in each group, n = 7 (*si‐Control*), 8 (*si‐VCL+TLN1*). E) Effect of *VCL+TLN1* knockdown on progesterone level in culture medium. n = 10 medium samples in each group. F) Experimental design of *G*, *H*. G) Effect of *VCL+TLN1* knockdown on the morphology and function of the in vivo corpus luteum. *Left*: representative photographs of ovarian sections. CL = corpus luteum, which are outlined by yellow frames. The CLs with cavities are outlined by green frames; F = follicle. Scale bar in the enlarged image: 100 µm. *Right*: statistics of the density of luteal cells, n = 22 (*si‐Control*), 21 (*si‐VCL+TLN1*) CLs. These CLs were observed from 4 and 7 biological independent ovaries, respectively. H) Effect of *VCL+TLN1* knockdown on progesterone level in serum. n = 7 serum samples in each group. Statistical significance was determined using two‐tailed unpaired Student's t test and Chi‐squared test, values were mean ± SD. ^***^ *p *< 0.001, ^****^ *p *< 0.0001. C and D were repeated independently five times, G was repeated two times, yielding consistent results.

Data in Figure 3 demonstrated that the disruption of focal adhesion assembly led to the failed mGC‐layer stiffening and subsequent release of mGCs from the manually punctured follicles. However, it is imperative to ascertain whether the disruption of focal adhesion assembly leads to the spontaneous release of mGCs from the post‐ovulatory follicle, akin to the cumulus‐oocyte complex (COC). To address this, real‐time filming of ovulation was conducted (**Figure** **4**A). We observed that the simultaneous knockdown of *VCL* and *TLN1* had no significant effect on ovulation rate (Figure 4B). However, compared to the control group where only the COC was expelled (Figure 4C/left, outlined by green frames), the mGC‐layer in the *si‐VCL+TLN1* group prominently protruded from the rupture site during COC expulsion (Figure 4C/left, outlined by yellow frames; Movie S7, Supporting Information). Remarkably, following complete COC expulsion, a substantial number of free cells flowed out from the rupture site (Figure 4C/left, outlined by red frames; Movie S7, Supporting Information), which were identified as mGCs through qRT‐PCR analysis of marker gene *Lhcgr* (Figure 4C/right). Subsequently, we evaluated the morphology and function of the newly formed corpus luteum at H40 (Figure 4D; Figure S2, Supporting Information). Compared to the control group, the knockdown groups *(si‐VCL*, *si‐TLN1*, and *si‐VCL+TLN1*) demonstrated reduced cell density and distinct cavitation in the corpus luteum (Figure 4D; Figure S2, Supporting Information). Moreover, the progesterone levels were decreased in the knockdown groups relative to the control groups (Figure 4E; Figure S2, Supporting Information). These results offer direct evidence supporting the critical role of focal adhesion‐mediated mGC‐layer stiffening in constraining mGCs within the post‐ovulatory follicle, facilitating their differentiation into a corpus luteum. Additionally, the downregulation of functional genes in luteal cells derived from residual mGCs was also observed following *VCL* and *TLN1* knockdown (Figure S2, Supporting Information). This suggests that disruption of focal adhesion could potentially disturb steroidogenesis in luteinizing mGCs. We further corroborated our findings through an in vivo knockdown experiment. Consistent with the abnormal phenotypes observed in vitro, concurrent knockdown of *VCL* and *TLN1* in the ovaries (Figure 4F) resulted in a notable decrease in cell density of the newly formed corpus luteum, along with the presence of cavities within some corpus lutea (Figure 4G). Moreover, the average serum progesterone content in the si‐*VCL+TLN1* mice was merely 30% of that observed in the control mice (Figure 4H).

### 2.5 Ovulatory Signal Stimulated Focal Adhesion Assembly and mGC‐Layer Stiffening by Activating the cAMP‐PKA‐CREB Cascade

**Figure 5:**
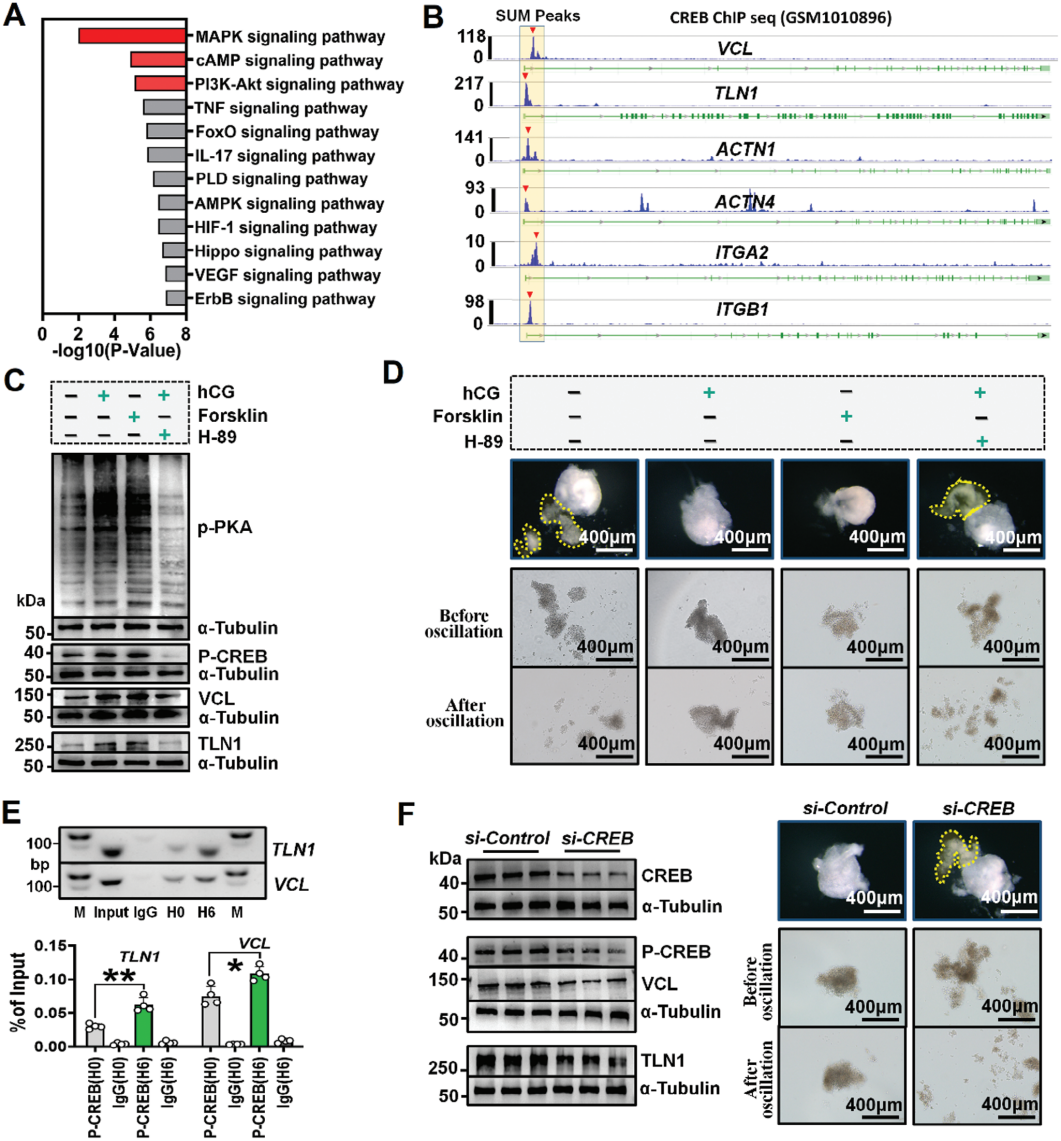
Ovulatory signal stimulated focal adhesion assembly and mGC‐layer stiffening by activating the cAMP‐PKA‐CREB cascade. A) KEGG analysis of the up‐regulated genes. B) Analysis of the binding of CREB to promoter of focal adhesion structural genes. The binding peaks of CREB are indicated by red triangles. C) Western blot assay of the protein contents of VCL and TLN1 following activation or inhibition of the cAMP‐PKA cascade. Original blots can be viewed in Figure S4D (Supporting Information). D) Effect of activating or inhibiting cAMP‐PKA cascade on the rigidity and escape capability of the mGC‐layer. The released mGC‐layers are outlined by yellow frames. E) ChIP‐qPCR assay for CREB binding to the promoters of *VCL* and *TLN1*. *Up*: electrophoretic images of PCR products. Input and IgG were used as positive and negative controls, respectively. Original gel images can be viewed in Figure S5 (Supporting Information). *Down*: statistical chart of qPCR assay. n = 4 mGC samples. F) Effect of *CREB* knockdown on focal adhesion assembly and “m GC‐layer stiffening”. *Left*: western blot assay of protein contents of VCL and TLN1 after *CREB* knockdown. Original blots can be viewed in Figure S4E (Supporting Information). *Right*: Effect of *CREB* knockdown on the rigidity and escape capability of the mGC‐layer. The released mGC‐layers are outlined by yellow frames. Statistical significance was one‐way ANOVA followed by Tukey's post hoc test, values were mean ± SD. *p < 0.05, **p < 0.01. C, D, and F were repeated independently three times, E was repeated two times, yielding consistent results.

To determine the signaling pathways involved in focal adhesion assembly and mGC‐layer stiffening, we analyzed genes upregulated by hCG (Figure 2C) using Kyoto Encyclopedia of Genes and Genomes (KEGG) analysis. This analysis revealed multiple signaling pathways, with the top three being the MAPK, cAMP, and PI3K‐AKT signaling pathway (**Figure** **5**A). In parallel, we used JASPAR (http://jaspar.genereg.net/) to predict the transcription factors binding to the promoters of focal adhesion structural genes in mice. Interestingly, CREB, a core transcription factor in cAMP signaling pathway, showed high binding scores (Figure S3A, Supporting Information). Moreover, by analyzing ChIP‐seq data of CREB derived from human embryonic stem cells (http://cistrome.org/db/#/, GSM1010896), we identified significant binding peaks of CREB in the promoters of six focal adhesion structural genes, including *VCL*, *TLN1*, *ACTN1*, *ACTN4*, *ITGA2*, and *ITGB1* (Figure 5B). Based on these observations, we hypothesized that the cAMP‐PKA‐CREB signaling cascade may play a crucial role in stimulating focal adhesion assembly and mGC‐layer stiffening.

To test this hypothesis, we conducted experiments using our follicle culture system. At H4, we observed that forskolin, an activator of adenylate cyclase, was sufficient to increase VCL and TLN1 protein levels by activating PKA‐CREB signaling even without hCG addition (Figure 5C). This activation resulted in mGC‐layer stiffening and prevented the escape of mGCs from punctured follicles at H6 (Figure 5D). Conversely, the use of H89, a PKA inhibitor, effectively suppressed PKA‐CREB signaling, impeding hCG‐induced increases in VCL and TLN1 levels (Figure 5C) and subsequent mGC‐layer stiffening (Figure 5D). Furthermore, ChIP‐qPCR assay unequivocally demonstrated that CREB directly interacts with the promoter regions of *VCL* and *TLN1*, and these interactions were notably enhanced after hCG stimulation (Figure 5E). Additionally, utilizing dual‐luciferase reporter assay, we identified the specific motifs within the promoters of *VCL* and *TLN1* that directly engage with CREB as CCAGGATGGCCTCAAACTTT and CAAGAGTGACATCATACACT, respectively (Figure S3B,C, Supporting Information). Lastly, we performed a knockdown of *CREB* expression in cultured follicles. Compared to the control group, the knockdown of *CREB* resulted in a significant reduction in VCL and TLN1 levels at H6, and more importantly, it led to the failure of mGC‐layer stiffening and subsequently the release of mGCs from punctured follicles (Figure 5F). Altogether, these results strongly confirm our speculation that the LH (hCG)‐cAMP‐PKA‐CREB signaling pathway is a key regulator of focal adhesion assembly and mGC‐layer stiffening.

## 3 Discussion

mGCs and cumulus cells, originating from the same progenitor cells, exhibit divergent fates post‐ovulation. While cumulus cells are released alongside the oocyte, mGCs remain sequestered within the post‐ovulatory follicle to differentiate into the corpus luteum. The mechanism that prevents mGC from escape the post‐ovulatory follicle has long been unclear. In our study, we introduce the concept of “mGC‐layer stiffening” to address this phenomenon, highlighting its induction by the ovulatory signals, specifically through the LH (hCG)‐cAMP‐PKA‐CREB pathway. Prior to the ovulatory signal stimulation, the mGC‐layer is in a flexible state, allowing for easy escape. However, following ovulatory signal stimulation, the mGC‐layer stiffens, impeding escape. Blockade of mGC‐layer stiffening results in mGCs release from the post‐ovulatory follicle and the formation of an abnormal corpus luteum characterized by low cell density or cavitation. Moreover, we observed mGC‐layer stiffening in goats as well (Figure 1G), indicating its evolutionary conservation among higher mammals.

Why does stiffening have the capacity to prevent mGCs from escaping the post‐ovulatory follicle? We hypothesize that the increased rigidity it imparts to the mGC‐layer is key. As illustrated in Movie S7 (Supporting Information), the rupture site on the follicle surface is considerably smaller than the size of COC, leading to COC deformation during exit. The maneuverability of the COC through the narrow rupture site depends on its flexibility, achieved through cumulus expansion. In contrast, the stiffened mGC‐layer lacks this flexibility, obstructing exit through the narrow opening. It is well‐documented that ECM stiffness plays a pivotal role in modulating mechanotransduction signaling pathways.^[^ ^23^, ^24^, ^25^ ^]^ Therefore, further investigations into the downstream signaling pathways triggered by mGC‐layer stiffening are warranted.

Our proposition suggests that focal adhesions, essential protein complexes mediating cell‐matrix interactions, serve as the structural basis for mGC‐layer stiffening. This assertion is supported by the failure in stiffening and ensuing mGC escape upon disruption of focal adhesions (Figures 3 and 4; Movie S7, Supporting Information). Notably, induction of focal adhesion assembly by ovulatory signals was also evident in specimens from goats, monkeys, and humans (Figure 2J–L), underscoring its evolutionarily conserved significance. Focal adhesions work in conjunction with the cytoskeleton, collectively transducing extracellular mechanical signals to modulate cellular processes such as proliferation, motility, deformability, substance transport, and organelle rearrangement. Prior studies have implicated cytoskeletal components, including intermediate filaments and microtubules, in the regulation of steroidogenic gene transcription, cholesterol transport, and ultimately steroidogenesis control.^[^ ^26^, ^27^, ^28^, ^29^ ^]^ Our study also demonstrates that disrupting focal adhesion results in a widespread decrease in the expression of steroidogenic genes in the luteal cells derived from residual mGCs (Figure S2, Supporting Information). Taken together, when discussing the luteal insufficiency observed in this study (Figure 4), it is essential to consider not only the escape of mGCs resulting from the disruption of focal adhesions but also the potential impact of such disruption on perturbing steroidogenesis in luteinizing mGCs by interfering with cytoskeletal function. Similarly, the observed cavitation in the corpus luteum of the *si‐VCL+TLN1* group could be attributed to a combined effect of mGC escape and compromised migration, given the essential roles of focal adhesions and the cytoskeleton in cell motility.^[^ ^30^ ^]^

The ovary is a mechanosensitive organ, where adhesion junctions play a significant role in folliculogenesis and ovulation by sensing and transmitting mechanical signals.^[^ ^31^, ^32^ ^]^ For instance, mGC‐specific knockout of N‐cadherin, a membrane component of intercellular junctions within mGC‐layer, resulted in impaired ovulation, COC expansion, and oocyte maturation.^[^ ^33^ ^]^ Despite this, research on the impact of focal adhesions on ovulation is currently limited. Our study found that disrupting focal adhesion assembly did not affect the ovulation rate (Figure 4B). However, Kitasaka et al. observed reduced ovulation count upon inhibiting focal adhesion kinase with Y3, indicating a potential role of focal adhesion‐mediated signals in ovulation.^[^ ^34^ ^]^ We speculate that this discrepancy may be attributed to two factors. First, the knockdown of *TLN1* and *VCL* might have minimal impact on FAK activity, as FAK primarily interacts with phosphatidylinositol biphosphate and paxillin, rather than directly with TLN1 and VCL.^[^ ^35^ ^]^ Second, despite achieving a 70% knockdown efficiency of TLN1 and VCL in the follicles (Figure S1, Supporting Information), the remaining intact focal adhesions (30%) could still be sufficient to support FAK activity for ensuring a normal ovulation count. In a recent study by Vann et al, mGC*‐PXN* KO mice were generated to specifically ablate paxillin, a structural protein of focal adhesion, in mGCs.^[^ ^36^ ^]^ Surprisingly, these mice displayed normal estrus cycles, ovulation, and fecundity, in contrast to the luteal dysfunction observed in our *si‐VCL+TLN1* mice (Figure 4). Vann et al. found that even in the absence of paxillin, VCL was still located at the cytomembrane, and mGC proliferation, migration, and attachment were unaffected. They thus proposed that paxillin's absence does not abrogate focal adhesion. However, we hypothesize that the divergence in phenotypes between mGC*‐PXN* KO and *si‐VCL+TLN1* mice may be attributed to the knockout strategy employed. One possibility is that the deletion of only exons 2–5 in mGC*‐PXN* KO mice, despite paxillin consisting of 12 exons, leads to the production of a truncated paxillin with functionality. Another possibility is that paxillin may have lesser significance compared to VCL and TLN1 in supporting the spatial structure of focal adhesion, resulting in limited impact on focal adhesion assembly. Experimental evidence is needed to validate these speculations.

**Figure 6:**
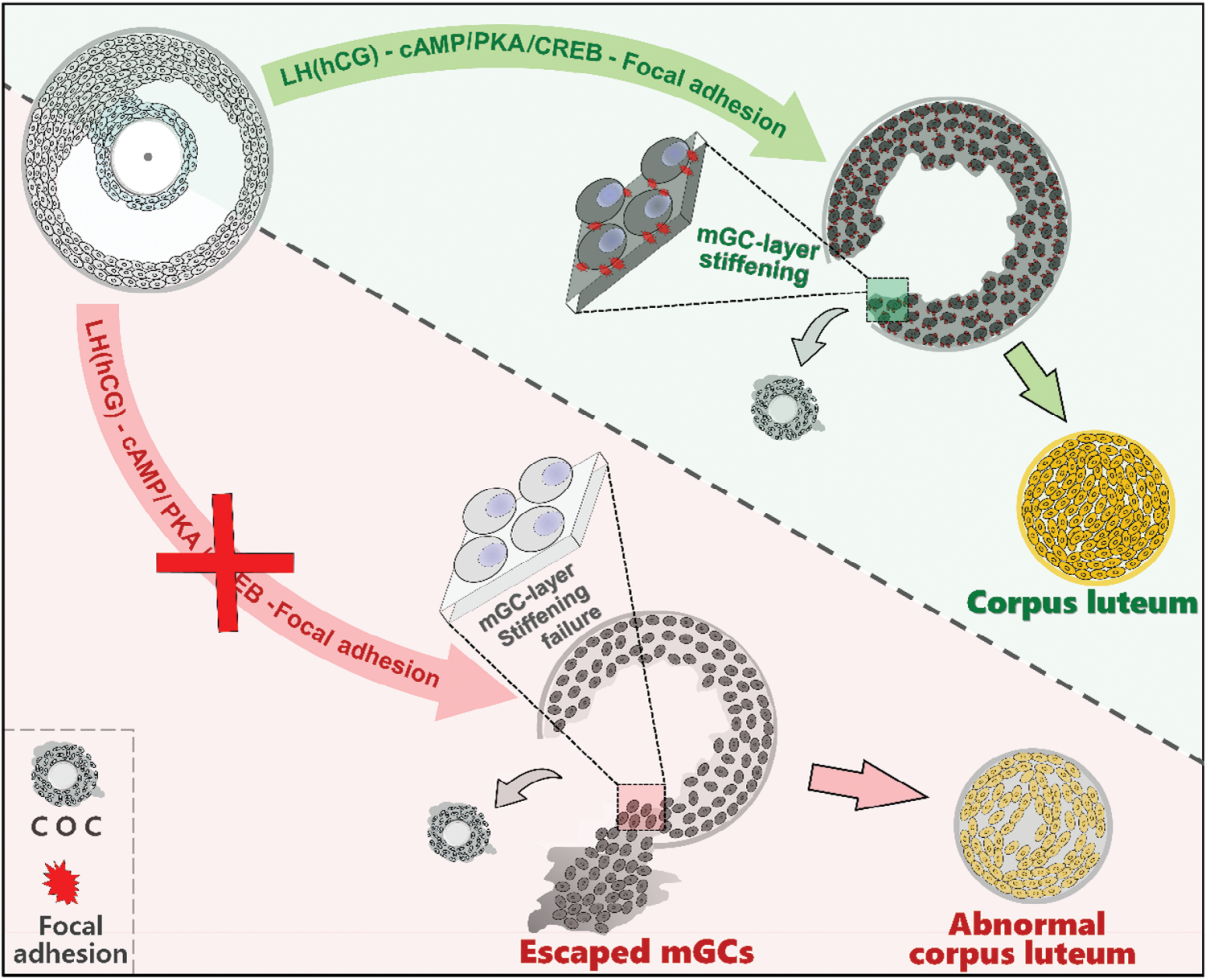
A diagram depicting mGC‐layer stiffening preventing mGCs from escaping a post‐ovulatory follicle. Ovulatory signals LH or hCG trigger mGC‐layer stiffening through a cAMP‐PKA‐CREB‐Focal adhesion cascade, confining mGCs within the ruptured follicle to form the corpus luteum. Inhibiting this cascade, either pharmacologically or through gene silencing, disrupts mGC‐layer stiffening, allowing mGCs to escape the follicle. This escape leads to an abnormal corpus luteum characterized by reduced cell density or cavities.

Overall, our study introduces the novel concept of “mGC‐layer stiffening” as a framework to elucidate the fundamental reproductive question of mGC retention within the post‐ovulatory follicle (**Figure** **6**). Moreover, it provides evidence of the evolutionary conservation of mGC‐layer stiffening, emphasizing its widespread importance across higher mammalian species. These findings provide valuable insights into the mechanism governing mGC confinement within the post‐ovulatory follicle, enriching our understanding of ovulation process and luteinization.

## 4 Experimental Section

### Animals

Kunming mice were purchased from the Experimental Animal Center of Huazhong Agricultural University (Wuhan, China). The mice were reared in an SPF laboratory animal house and maintained at a constant temperature of 22 ± 2 °C, with 12 h light‐dark cycles (lights on from 7:00 to 19:00). They were allowed to access food and water ad libitum. The Hainan black goats, on the other hand, were raised at the Yazhou Beiling Black Goat Farmers' Professional Cooperative in Sanya, China. All experiments and handling of mice and goats were conducted following the guidelines of the respective animal experimental institutions. Prior approval from the Institutional Animal Ethics Committee of Huazhong Agricultural University was obtained, with the approved protocol number being HZAUMO‐2020‐0103 (mouse); HZAUGO‐2020‐004 (goat).

### Analysis of Spatial Transcriptomics and RNA‐Seq

The spatial transcriptome data of mouse ovaries, contributed by Mantri et al,^[^ ^16^ ^]^ was obtained from the Gene Expression Omnibus database (Login Number: GSE240271). The bead barcode location files corresponding to spatial transcriptomics datasets, processed spatial metadata, and cell annotations files, were sourced from GitHub (https://github.com/madhavmantri/mouse_ovulation). The assignments of distinguishing the pre‐ovulatory follicles and labeling the mGCs have been completed by Mantri et al. the identity of mGCs were re‐confirmed within the pre‐ovulatory follicles based on the expression abundance of marker genes *Lhcgr* and *Adamts1*. Subsequently, barcode data was extracted for these mGCs and utilized the scanpy analysis package (v1.9.5)^[^ ^37^ ^]^ to calculate the total counts of all genes in mGCs, along with the total number of mGCs (n_cells_total_counts) within each pre‐ovulatory follicle. These values were then used to calculate the mean counts for each gene using the formula: mean_counts = total_counts/n_cells_total_counts. Genes with mean counts > 0 were selected for bioinformatics analysis. Transcriptome data for monkey and human were obtained from the Gene Expression Omnibus database, with login numbers GSE22776 and GSE133868, respectively.^[^ ^17^, ^18^ ^]^ PCA analysis was employed to assess the transcriptomic differences among mGCs from different pre‐ovulatory follicles. Differentially expressed genes were identified using DESeq2 software, with a significance threshold of P‐value <0.05. The upregulated genes were then subjected to GO analysis using the DAVID database (https://david.ncifcrf.gov/tools.jsp), and KEGG analysis using the KOBAS database (http://kobas.cbi.pku.edu.cn/home.d).

### Follicle Culture

Small follicles measuring 180–200 µm in diameter were isolated from ovaries using 33‐gauge microneedles (KONSFI, China). The isolated follicles were cultured in 96‐well plates (BKMAM, China) coated with mineral oil (50 µL) (Sigma‐Aldrich, USA) and placed in a 37 °C incubator with 5% CO_2_. The culture medium for maturation consisted of ɑ‐MEM (Gibco, USA) supplemented with ITS‐G (1%) (Macklin, China), FBS (5%) (Serana, Germany), FSH (10 mIU mL^−1^, NSHF, China), and penicillin/streptomycin (100 U mL^−1^, Servicebio, China). After 96 h of culture, follicles reaching the pre‐ovulatory stage (500‐550 µm) were transferred to ovulation‐inducing medium and cultured for up to 16 h. The ovulation‐inducing medium contained ɑ‐MEM supplemented with ITS‐G (1%), FBS (5%), FSH (10 mIU mL^−1^), hCG (1.5 IU mL^−1^, NSHF, China), EGF (10 ng mL^−1^, PeproThec, USA), D‐Glucose (5 mg mL^−1^, MCE, USA), and penicillin/streptomycin (100 U mL^−1^). In experiments studying the signaling pathway, Forsklin (MCE, USA) and H‐89 (MCE, USA) dissolved in DMSO were added to the ovulation induction solution at concentrations of 20 and 50 µM, respectively. After ovulation, the post‐ovulatory follicles were transferred to luteal culture medium and cultured for up to 24 h. The luteal culture medium consisted of ɑ‐MEM supplemented with ITS‐G (1%), FBS (5%), FSH (10 mIU mL^−1^), hCG (1.5 IU mL^−1^), EGF (10 ng mL^−1^), Prolactin (1 ng mL^−1^, MCE, USA), Cholesterol (10 µM) (MCE, USA), and penicillin/streptomycin (100 U mL^−1^).

### Superovulation

To stimulate follicle growth to the pre‐ovulatory stage in weaned juvenile mice, an injection of PMSG (5 IU) (Ningbo Sansheng Biological Technology, China) was administered. After 48 h, ovulation and luteinization were triggered by injecting hCG (5 IU) (Ningbo Sansheng Biological Technology, China). In goats, vaginal plugs containing progesterone (Zoetis Australia Pty Ltd, New Zealand) were pre‐inserted to synchronize their estrus cycle. For superovulation induction, the goats were injected with follicle‐stimulating hormone (40 IU) (Ningbo Sansheng Biological Technology, China) seven times at 12 h intervals, starting 84 h before the removal of the plugs. At the time of plug withdrawal, PMSG (240 IU) was injected. After an additional 14 h, LH (100 IU) (Ningbo Sansheng Biological Technology, China) was injected, and the mGCs clumps were obtained for rigidity determination by puncturing the pre‐ovulatory follicles 0 and 10 h after LH injection.

### Determination of mGC‐Layer Escape Capability and Rigidity

The ovaries or cultured follicles were punctured with a microneedle at the designated time point to assess the escape capability and rigidity of the mGC‐layer. For the escape capability determination experiment, to ensure the reproducibility of experimental observations, all samples were punctured by the same operator in a single trial. The operator followed a standardized procedure during puncture, initiating with a single piercing of the follicular wall using the needle tip, followed by pressing on the follicle surface with the same force and frequency using the non‐needle tip, while closely monitoring and recording the escape of mGCs. Furthermore, to prevent experimental bias, the operator remained blinded to the group allocation when handling different follicles.

In the rigidity determination experiment, the follicle was punctured and pressed following the same procedure described above. For the H6 and H10 follicles, isolation of mGC is challenging. Consequently, these follicles underwent repeated punctures, following which mGC clumps were meticulously extracted through a combination of pressing and pulling actions using the needle. Subsequently, the released mGC clumps were aspirated using pre‐prepared large‐bore glass capillaries, transferred to a 12‐well plate containing DMEM/F12 buffer (Gibco, USA), and placed in a thermostatic shaker (Leopard, China) for 1 min of mechanical oscillation. The oscillations were conducted at a parameter of 700 rpm at 37 °C. The process was documented and visually captured using a stereo microscope (Olympus Corporation, Japan, SZX16). As for the collection protocol for goat mGC clumps, surgical excision of the ovaries was performed, followed by transfer to a 60 mm culture dish. Pre‐ovulatory follicles protruding from the ovarian surface were incised with a surgical blade to release the mGC clumps, with subsequent steps mirroring those described for mice.

### Purification of mGCs and Cumulus Cells

14 h after hCG supplementation, the ovulated COCs from cultured follicles were collected under a stereomicroscope (Olympus, Japan). The COCs were then treated with hyaluronidase (Sigma‐Aldrich, USA) to dissociate cumulus cells from the oocyte. After removing the oocytes, purified cumulus cells were isolated and utilized as the negative control for Figure 4C. Subsequently, post‐ovulatory follicles were punctured with a 1 mL syringe to extract mGC clumps, which served as the positive control.

### Live Recording of Ovulation

The pre‐ovulatory follicles were cultured in a 96‐well plate with ovulation‐inducing medium (60 µL) per well. The plate was then placed in a 37 °C incubator with 5% CO_2_. After 8 h of culture, the plate containing the follicles was transferred to a live‐cell imaging system (Agilent BioTek Cytation 5, USA) to capture images at 6‐min intervals during ovulation. Subsequently, all the images were compiled to create a comprehensive video.

### RNA Interference

Lentivirus‐mediated RNA interference was used to inhibit the expression of target genes in follicles or ovaries. Briefly, PLKO.1‐EGFP‐PURO plasmid (Genecreate, China) was utilized to construct interference vectors. Small interfering RNAs (siRNAs) targeting *VCL*, *TLN1*, and *CREB* were synthesized by Genepharma (China), with the following targeted sequences: *VCL –* 5′‐ccacgatgaagctcggaaatg‐3′, *TLN1 –* 5′‐gcccattgtaatctctgctaa‐3′, *CREB –* 5′‐cagcagctcatgcaacatcat‐3′. The common negative siRNA was purchased from Sigma‐Aldrich (USA). Lentiviruses were produced in 293 T cells (ATCC, USA) by co‐transfecting the interference vector (4.8 µg), pMD2.G (2.4 µg), and pSPAX2 (3.6 µg). After 48 h, the viral supernatants were harvested, centrifuged, and filtered through 0.45 µm polyvinylidene fluoride (PVDF) membranes (Sigma, USA). For knocking down gene expression in the cultured follicle, the small follicles were isolated and cultured in a 96‐well plate with maturation medium (60 µL). After a 48 h culture period, the follicles were transferred to maturation medium supplemented with 10% lentivirus (titer: 1.25 × 10^7^ viral particles mL^−1^). Following an additional 48 h plasmid transfection period, follicles reaching the pre‐ovulatory stage were transferred to ovulation‐inducing medium and cultured for up to 16 h, with real‐time ovulation recorded. Subsequently, the post‐ovulatory follicles were incubated in luteal culture medium for subsequent luteal function assays. For knocking down gene expression in the ovaries, 15‐day‐old mice were anesthetized. Subsequently, lentivirus with a titer of 1.25 × 10^9^ viral particles mL^−1^ (2.5 µL) was injected beneath the ovarian bursa using a syringe (10 µL) (Hamilton, Switzerland) and a 33‐gauge Small Hub RN Needle (Hamilton, Switzerland). Follow‐up experiments on these mice were conducted 5 days after plasmid transfection.

### qRT‐PCR Analysis

Total RNA was extracted from the collected samples using the Trizol reagent. Reverse transcription was performed using the PrimeScript RT reagent kit (Takara, Japan). The qRT‐PCR was conducted using a CFX384 Real‐Time PCR System (Bio‐Rad, USA). The reaction mixture comprised of SYBR Green (5 µL, Biosharp, China), complementary DNA template (2 µL), 250 nM each of the forward and reverse primers, and ddH_2_O to make a total volume of 10 µL. The reaction conditions were as follows: initial denaturation at 95 °C for 10 min, followed by 35 cycles of denaturation at 95 °C for 10 s, and annealing/extension at 60 °C for 30 s. A final step included a melting curve analysis ranging from 60 °C to 95 °C, with a 0.5 °C increment every 5 s. Gene expression levels were normalized using *ACTB*, and the relative RNA quantification was determined using the comparative 2^−△△Ct^ method. The primer sequences used for PCR amplification are provided in Table S1 (Supporting Information).

### Frozen Section and H&E Staining

The ovaries or cultured corpora lutea were collected as per experimental requirements and embedded in an OCT embedding medium (Sakura, USA) for subsequent processing. The embedded tissues were flash‐frozen in liquid nitrogen for 1 min and then sectioned into 6 µm‐thick slices using a frozen microtome (Leica, Germany). The slices were stained with hematoxylin and eosin (Servicebio, China) and imaged using a microscope (Olympus, Japan). The density of luteal cells was quantified using ImageJ software (V1.49) following the software's operation manual. The procedure involved importing the image; measuring the areas of corpus luteum and cavities within it; converting the image to black and white; enhancing cell nuclei by adjusting the contrast threshold, and counting the number of cell nuclei using the Analyze Particles plugin. The luteal cell density was calculated using the formula: luteal cell density = number of luteal cells/(corpus luteum area – cavity area).

### ChIP‐qPCR Assay

ChIP‐qPCR was employed to assess the abundance of CREB binding in the promoter regions of *VCL* and *TLN1*. The isolated mGC samples were fixed in 10 mL of DMEM/F‐12 supplemented with formaldehyde (1%) (Cell Signaling Technology, USA) for 10 min at room temperature with rotation. The reaction was then quenched by adding 1 mL of 1.5 M glycine and rotating for an additional 5 min at room temperature. The samples were transferred into a 1.5 mL centrifuge tube (Axygen, USA) containing PBS for wash, and then lysed in cytomembrane lysis buffer at 4 °C for 15 min with mixing every 5 min. The buffer contains HEPES (10 mm) (Sigma‐Aldrich) at pH 7.9, IGEPAL‐CA630 (0.5%) (Sigma‐Aldrich, USA), MgCl_2_ (1.5 mm) (Sigma‐Aldrich, USA), KCl (10 mm) (Sigma‐Aldrich, USA), and a protease inhibitor cocktail (Sigma‐Aldrich, USA). Following this, the samples were further lysed in nuclear lysis buffer, containing SDS (1%) (Sigma‐Aldrich, USA), EDTA (10 mm) (Sigma‐Aldrich, USA), Tris (50 mm) (Sigma‐Aldrich, USA) at pH 8.1, and a protease inhibitor cocktail, for 15 min at 4 °C. Finally, the chromatin was sonicated using an ultrasonic disintegrator (Bioruptor PLUS, Belgium) to fragment the DNA into sizes ranging from 100 to 500 bp. The immunoprecipitation (IP) experiments were performed using the Magna ChIP A/G Chromatin Immunoprecipitation Kit (Merck, USA). In brief, the supernatant obtained from sonicated chromatin was diluted with ChIP IP buffer. Immunoprecipitation was performed by adding P‐CREB antibody (2 mg) (9198S, CST, USA) to protein A/G Dynabeads (Life Technologies, USA) and incubating the mixture overnight at 4 °C. The antibody‐bound beads were then washed, and the DNA‐protein complexes were eluted and subjected to reverse crosslinking. DNA purification was carried out using the QIAquick PCR Purification Kit (Qiagen, Germany). The amplification products were visualized by agarose gel electrophoresis (80 V, 80 mA, 75 min). The primers designed for amplifying the promoter regions of *VCL* and *TLN1* were based on the sequences of the CREB binding motifs. The specific primer sequences can be found in the Table S1 (Supporting Information).

### Western Blot

Total proteins were extracted with RIPA lysis buffer (ComWin Biotech, China) supplemented with protease and phosphatase inhibitors (ComWin Biotech, China) and PMSF (Solarbio, China). Protein contents was determined using the BCA Protein Assay Kit (Servicebio, China). The proteins were separated by polyacrylamide gel electrophoresis and transferred onto a polyvinylidene fluoride membrane. Following transfer, the membrane was blocked with skim milk powder (5%) (Nestle, Switzerland) at room temperature and then incubated overnight at 4 °C with the appropriate primary antibodies, including: VCL (1:1000 dilution, A2752, Abclonal, China), TLN1 (1:1000 dilution, A4158, Abclonal, China), phosphor‐PKA (1:1000 dilution, 5661S, CST, USA), CREB (1:1000 dilution, A11064, Abclonal, China), phospho‐CREB (1:1000 dilution, 9198S, CST, USA) and α‐tubulin (1:1000 dilution, GB15201, Servicebio, China). The membrane was subsequently washed three times with TBST (Solarbio, China) and incubated with the appropriate HRP‐conjugated secondary antibodies (goat anti‐rabbit secondary antibody, 1:4000 dilution, BF03008; goat anti‐mouse secondary antibody, 1:4000 dilution, BF03001, Biodragon‐immunotech, China) for 1 h at room temperature. After washing with TBST, the protein bands were visualized using an ECL chemiluminescent reagent kit (Servicebio, China). Images were captured using a Chemiluminescence imager (Image Quant LAS4000 mini). The protein levels were normalized to the expression of the housekeeping protein α‐tubulin.

### Immunofluorescence Staining

The isolated follicles were embedded in OCT (Sakura, USA) and frozen, and then sectioned into 5 µm thick slices. The sections were rewarmed and fixed, followed by high‐temperature antigen retrieval at 95–98 °C for 25 min using an antigen retrieval buffer (5%) (Servicebio, China). Next, the sections were blocked with goat serum (10%) (Boster, China) for 1 h at room temperature. Dual‐staining of VCL and TLN1 was conducted using a Three‐color Fluorescence kit (Recordbio Biological Technology, China) based on Tyramide Signal Amplification (TSA) technology. Briefly, following antigen blocking, the sections were incubated overnight at 4 °C with VCL antibody (1:400 dilution, 66305‐1‐Ig, Proteintech, USA). After rinsing with PBS, the sections were incubated with an HRP‐conjugated secondary antibody working solution (RCB054, Recordbio Biological Technology, China) at 37 °C for 50 min. Subsequently, the sections were treated with the fluorescent dye TYR‐570 (Recordbio Biological Technology, China) for 5 min, followed by a heat treatment to eliminate nonspecific dye binding. The same protocol was applied for TLN1 detection using the primary antibody against TLN1 (1:300 dilution, 14168‐1‐AP, Proteintech, USA) and fluorescent dye TYR‐690 (Recordbio Biological Technology, China) for TLN1 staining. Additionally, cell nuclei were stained with DAPI (Biosharp, China). Following another round of washing, the sections were imaged using an LSM800 confocal microscope system (Zeiss, Germany) and the resulting images were analyzed using Zen 2.3 lite software.

### Luciferase Reporter Assay

To construct the reporter vectors, the promoter regions of *VCL* and *TLN1* were amplified and inserted into the PGL3‐Basic luciferase reporter vector (Promega, USA) using the ClonExpress Ultra One Step Cloning Kit (Vazyme, China). Concurrently, the promoter regions of of *VCL* and *TLN1* containing single base mutations were inserted into the PGL3‐Basic luciferase reporter vector using the Mut Express II Fast Mutagenesis Kit (Vazyme, China). For the construction of the *CREB* over‐expression vector, the full‐length coding sequence of *CREB* was amplified and inserted into the pcDNA3.1 plasmid (Addgene, USA). HEK293T cells were seeded in a 24‐well plate and incubated for 24 h. Then, the *CREB* over‐expression vector, the constructed pGL3‐Basic reporter vectors, and the pRL‐TK vector (Promega, USA) were co‐transfected into the cells using the jetPRIME transfection reagent (Polyplus‐transfection, France) at a ratio of 96: 96: 1. After 24 h of transfection, the cells were lysed in lysis buffer (100 µL) and subjected to promoter activity assay using the dual luciferase reporter assay system (Promega, USA). The luciferase enzymatic activity was measured using a PE Enspire Multilabel Reader (PerkinElmer, USA). The primers used in this experiment are listed in Table S1 (Supporting Information).

### Hormone Determination

The levels of progesterone in serum and culture medium were quantified using radioimmunoassay. Briefly, sera were obtained by centrifuging whole blood at 3000 rpm for 10 min and stored at −20 °C. Culture medium was directly collected and stored at −20 °C. Detection kits purchased from the Bioengineering Institute (Nanjing, China) were utilized for the analysis, which was conducted by the North Institute of Biological Technology (Beijing, China).

### Statistics Analysis

Statistical analyses were using GraphPad Prism 10.0 (GraphPad). Data were expressed as the mean ± SD. Two‐tailed unpaired Student's t test and one‐way analysis of variance followed by Tukey's post hoc test were used to analyze the statistical significance between two groups and among multiple groups, respectively. Chi‐squared test was used in the comparison between the percentages. The statistical significance was set at *P*‐value <0.05.

## Conflict of Interest

The authors declare no conflict of interest.

## Author Contributions

X.W., J.L., and H.S. contributed equally to this work. C.H. conceived, designed, funded, supervised, and conducted the experiments, and wrote the manuscript; X.W., J.L., and H.S. anticipated in experiment design and conduction, data analysis, and manuscript preparation; Y.Z., W.K., and H.W assisted with sample collection and experiments conduction; G.L. and X.L. provided advice through project implementation and improved the manuscript. All authors approved the final version.

## Supporting information

Supporting Information

Supplemental Movie1

Supplemental Movie2

Supplemental Movie3

Supplemental Movie4

Supplemental Movie5

Supplemental Movie6

Supplemental Movie7

## Data Availability

The data that support the findings of this study are available from the corresponding author upon reasonable request.
