# Supplementary material for: Granulosa Cell‐Layer Stiffening Prevents Escape of Mural Granulosa Cells from the Post‐Ovulatory Follicle
Source: Adv Sci (Weinh). 2024 Jul 1;11(33):2403640. doi: 10.1002/advs.202403640 (PMC11434234; doi:10.1002/advs.202403640)
Supplement: Supplementary file 1 — Supporting Information [file ADVS-11-2403640-s001.docx]

**Supplementary data**

**Granulosa cell-layer stiffening prevents escape of mural granulosa cells from the post-ovulatory follicle**

**
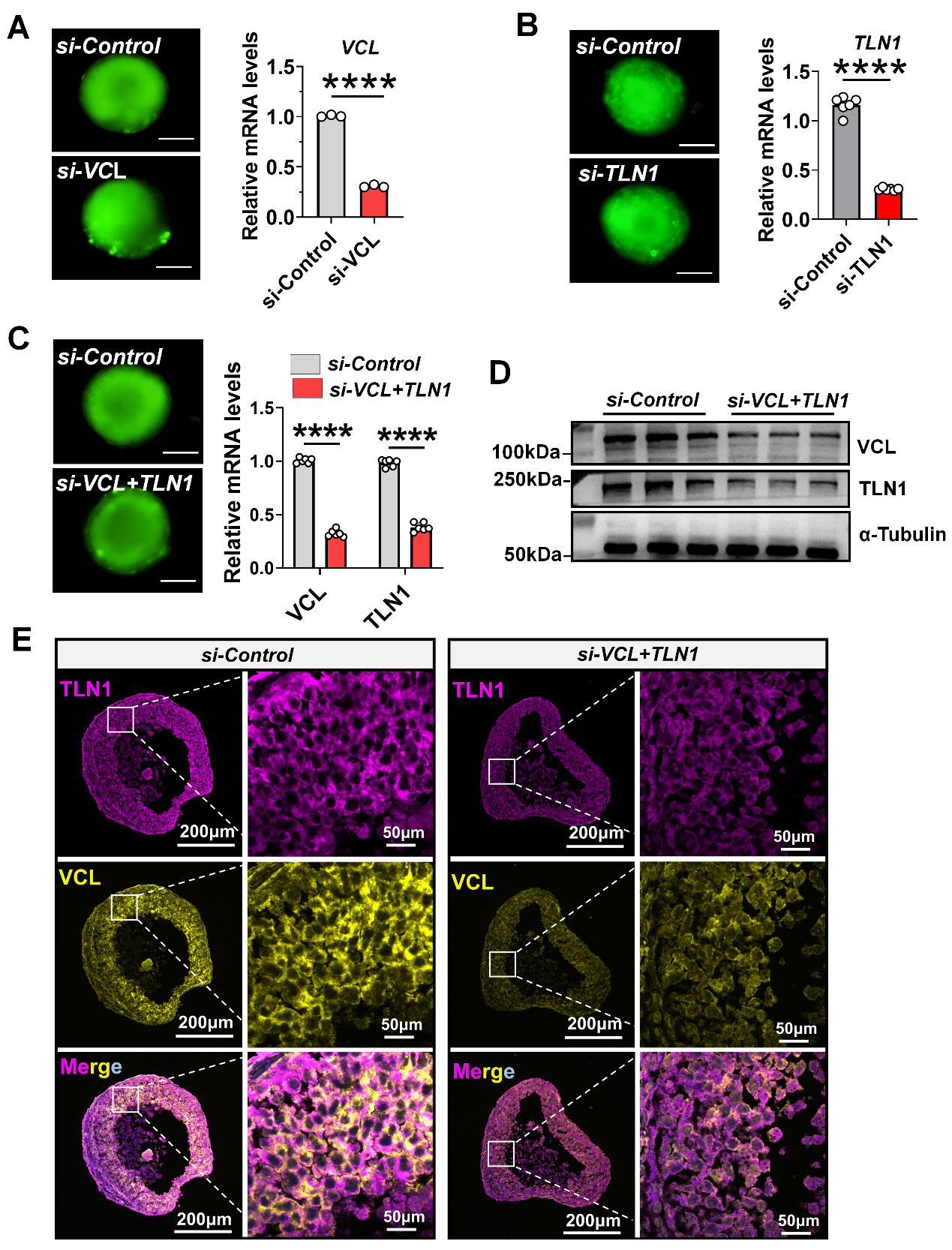
**

**Figure S1.** **Efficiency analysis of RNA interference (related to Figure 3).** A) qRT-PCR analysis of the efficiency of *VCL* interference. Green fluorescence indicates successful transcription of the interfering plasmids in follicles, Scale bar: 200 µm, n = 3 follicular samples. B) qRT-PCR analysis of the efficiency of *TLN1* interference. Green fluorescence indicates successful transcription of the interfering plasmids in follicles, Scale bar: 200 µm, n = 6 follicular samples. C) qRT-PCR analysis of the efficiency of *VCL*+*TLN1* interference. Green fluorescence indicates successful transcription of the interfering plasmids in follicles, Scale bar: 200 µm, n = 6 follicular samples. D) Western blot analysis of the efficiency of *VCL+TLN1* interference in cultured follicles. n = 3 follicular samples. Original blots can be viewed in Figure. S4C. E) Dual fluorescent staining analysis of the co-localization signal of VCL and TLN1 in mGC-layer after *VCL* and *TLN1* knockdown. The follicular samples for qRT-PCR assay and western blot assay were prepared by lysis of 15 and 30 follicles, respectively. The commercial scrambled shRNA was used as *si-Control*. Significance was determined using two-tailed unpaired Student’s t-test, with value presented as mean ± SD. Significant differences were denoted by ****P<0.0001. A, B and C were repeated independently three times, E was repeated two times, yielding consistent results.


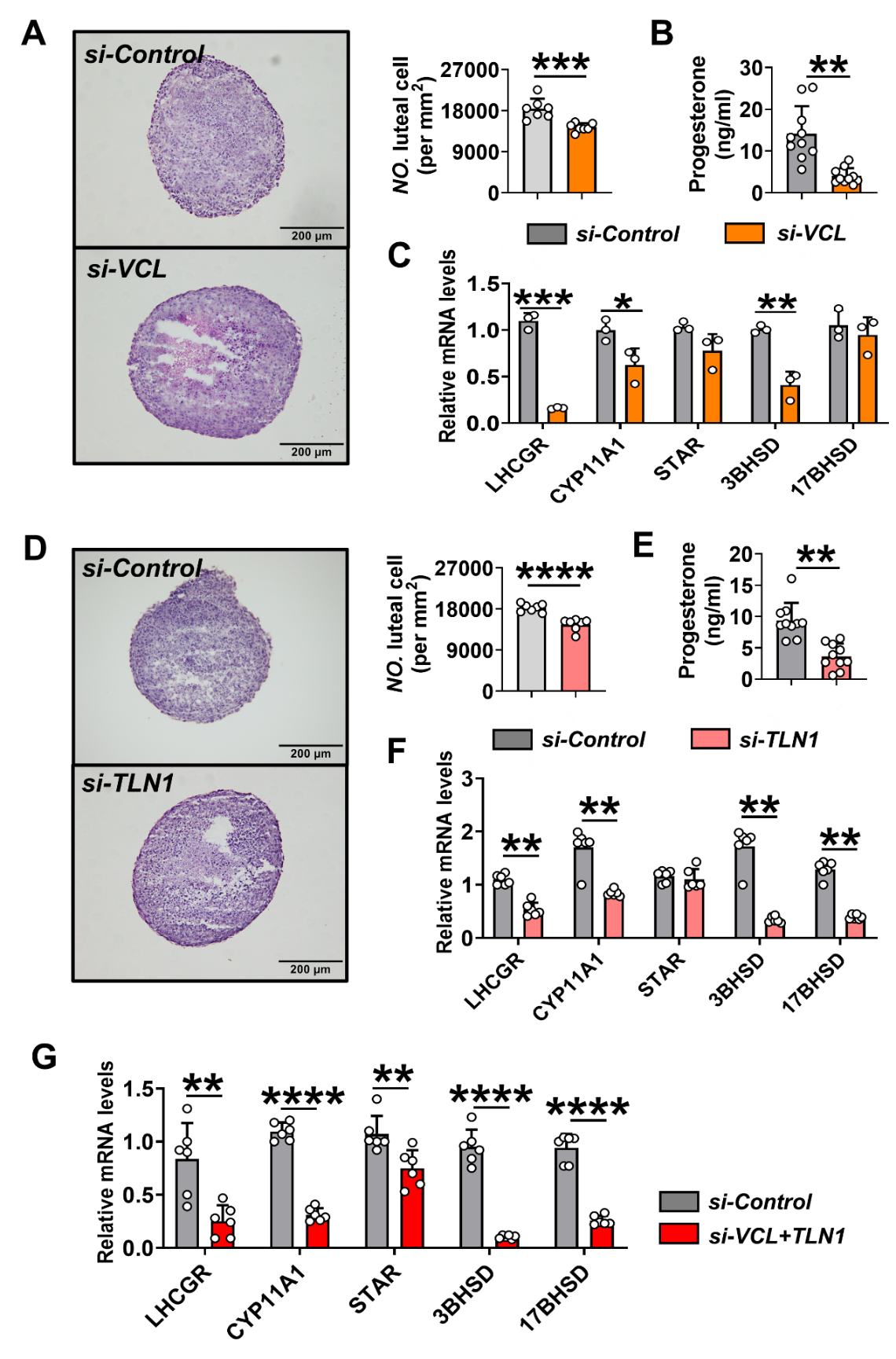


**Figure S2. Effect of *VCL* and *TLN1* knockdown on the morphology and function of culture corpus luteum (related to Figure 4).** A) Effect of *VCL* knockdown on the luteal cell density. *Left:* representative photographs of corpus luteum sections in each group, scale bar: 200 µm; *right*: statistics of the density of luteum cells in each group (n= 7). B) Effect of *VCL* knockdown on progesterone level in culture medium (n=10). C) Change in the expression of luteal functional genes after *VCL* knockdown (n=3). D) Effect of *TLN1* knockdown on the luteal cell density. *Left:* representative photographs of corpus luteum sections in each group, scale bar: 200 µm; *right:* statistics of the density of luteum cells in each group (n = 7). E) Effect of *TLN1* knockdown on progesterone level in culture medium (n=10). F) Change in the expression of luteal functional genes after *TLN1* knockdown (n = 6). G) Change in the expression of luteal functional genes after *VCL+TLN1* knockdown (n = 6). Significance was determined using two-tailed unpaired Student’s t-test, values were mean ± SD. Significant differences were denoted by *P<0.05, **P<0.01, ***P<0.001, ****P<0.0001. The experiments were repeated three times, yielding consistent results.


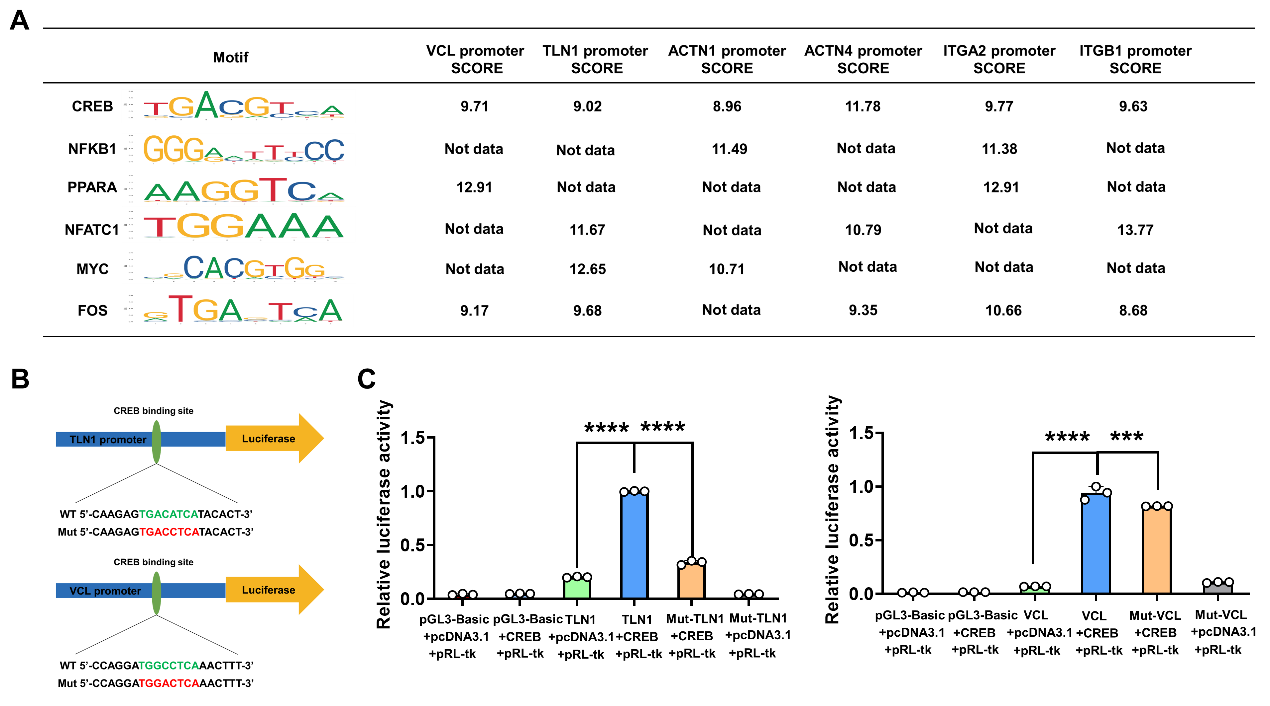


**Figure S3. Analysis of motifs in promoters of focal adhesion structural genes and dual-luciferase reporter assay (related to Figure 5).** A) The analysis based on JASPAR database showed that a CREB binding motif is present in the promoter regions of focal adhesion structural genes. B) Construction of luciferase reporters of WT, *VCL/TLN1* and Mut *VCL/TLN1*. C­­) WT or Mut *VCL*, *TLN1* Promoter Luc was measured in HEK293T cells with *CREB* overexpression. The empty pcDNA3.1 vector was used as a negative control (n = 3). Statistical signiﬁcance was determined using two-tailed unpaired Student’s t test, values were mean ± SD. Significant differences were denoted by ***P<0.001, ****P<0.0001. The experiments of C were repeated three times, yielding consistent results.

**
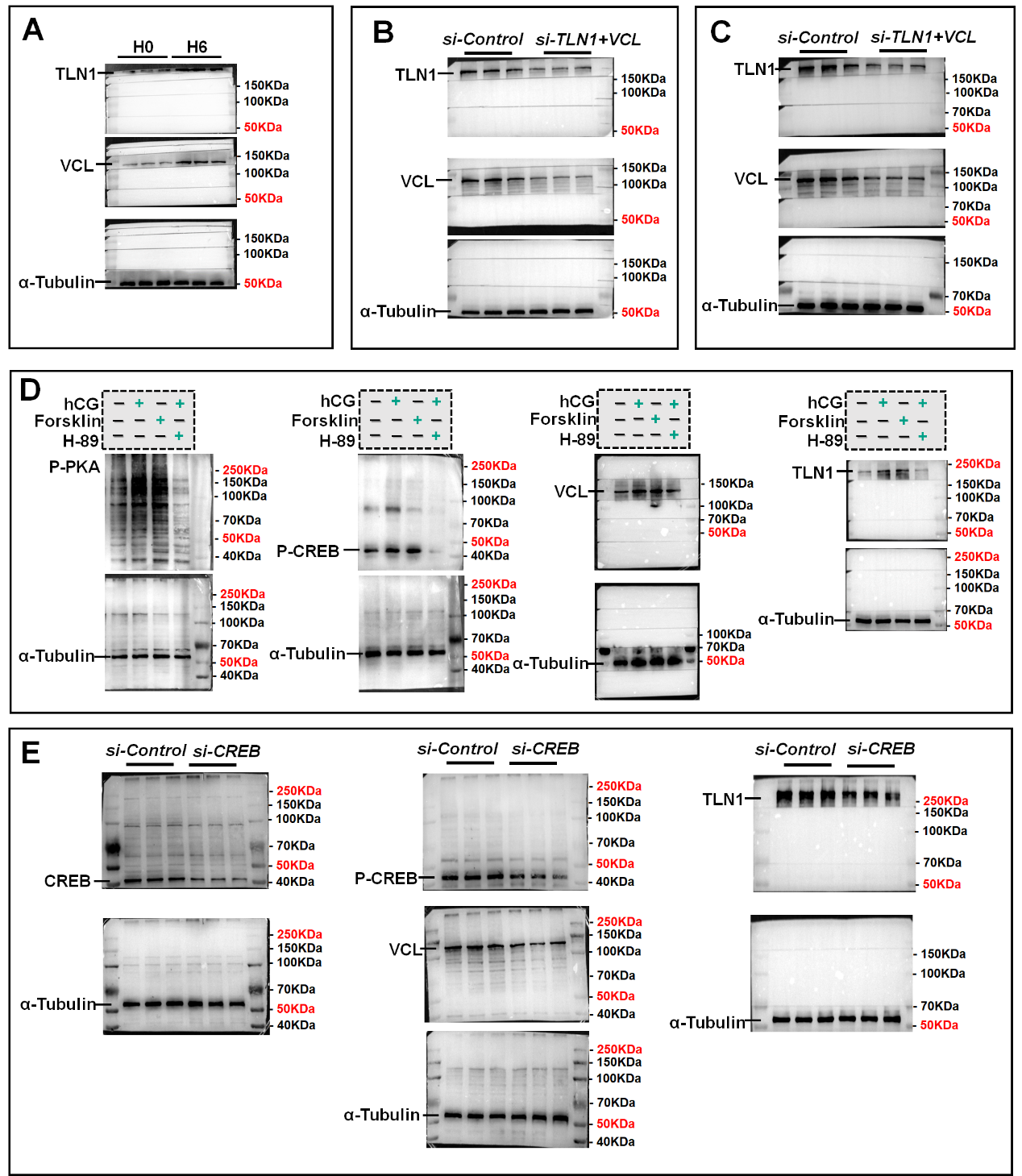
**

**Figure S4. Full western blots.** A) Western blot analysis of VCL and TLN1 protein levels post-hCG injection (related to Figure 2H). B) Western blot analysis of VCL and TLN1 protein levels in ovaries after RNA interference (related to Figure 3F). C) Western blot analysis of VCL and TLN1 protein levels in cultured follicles after RNA interference (related to Figure S1D). D) Western blot analysis of VCL and TLN1 protein levels following activation or inhibition of the cAMP-PKA cascade (related to Figure 5C). E) Western blot analysis of VCL and TLN1 protein levels after CREB knockdown (related to Figure 5F).


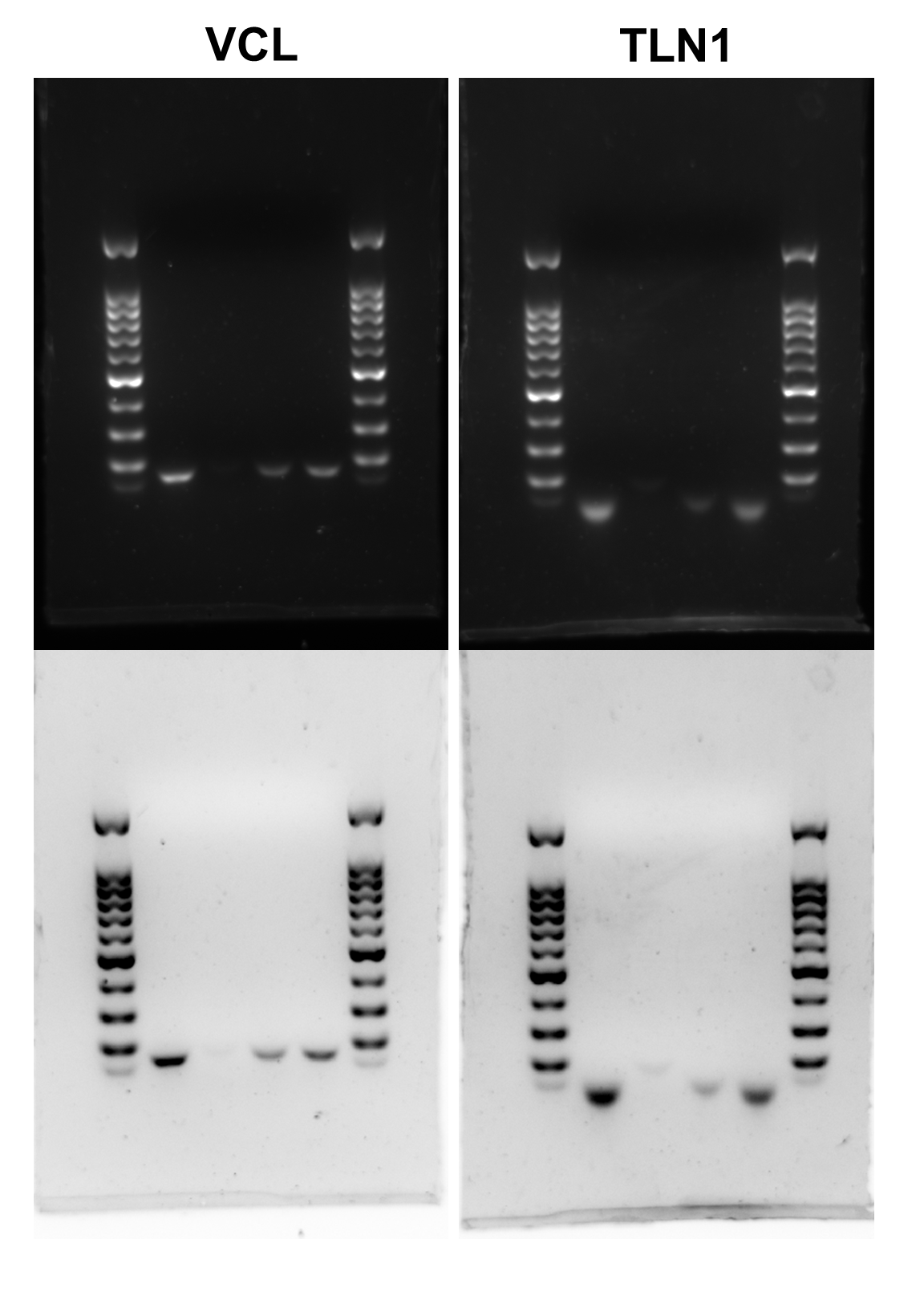


**Figure S5. Original agarose gel eletrophoresis in Figure 5E.**

**Movie S1.** The movie illustrates the reduced capability of mGCs to release from the punctured follicles following hCG supplementation.

**Movie S2.** The movie illustrates the reduced capability of mGCs to release from the punctured ovaries after hCG injection.

**Movie S3.** The movie visualizes the augmented rigidity of mGC-layer following hCG injection.

**Movie S4**. The movie showcases the compromised retention of mGCs within the follicle post *VCL* knockdown.

**Movie S5.** The movie showcases the compromised retention of mGCs within the follicle post *TLN1* knockdown.

**Movie S6.** The movie showcases the compromised retention of mGCs within the follicle post *VCL+TLN1* knockdown.

**Movie S7.** The movie captures the spontaneous release of mGCs from the post-ovulatory follicle in *si-VCL+TLN1* group.

**Table S1. The primers used for qPCR, ChIP-qPCR and Luciferase reporter**

| Gene | Primer sequence (5'-3') |
| --- | --- |
| *Tjp1-mouce* | Forward: GCCGCTAAGAGCACAGCAA |
|  | Reverse: TCCCCACTCTGAAAATGAGGA |
| *Tjp2-mouce* | Forward: ATGGGAGCAGTACACCGTGA |
|  | Reverse: TGACCACCCTGTCATTTTCTTG |
| *Tjp3-mouce* | Forward: TCGGCATAGCTGTCTCTGGA |
|  | Reverse: GTTGGCTGTTTTGGTGCAGG |
| *Cldn1-mouce* | Forward: GGGGACAACATCGTGACCG |
|  | Reverse: AGGAGTCGAAGACTTTGCACT |
| *Cldn2-mouce* | Forward: CAACTGGTGGGCTACATCCTA |
|  | Reverse: CCCTTGGAAAAGCCAACCG |
| *Cldn3-mouce* | Forward: ACCAACTGCGTACAAGACGAG |
|  | Reverse: CAGAGCCGCCAACAGGAAA |
| *Cldn4-mouce* | Forward: GTCCTGGGAATCTCCTTGGC |
|  | Reverse: TCTGTGCCGTGACGATGTTG |
| *Cldn6-mouce* | Forward: ATGGCCTCTACTGGTCTGCAA |
|  | Reverse: GCCAACAGTGAGTCATACACCTT |
| *Ocln-mouce* | Forward: TTGAAAGTCCACCTCCTTACAGA |
|  | Reverse: CCGGATAAAAAGAGTACGCTGG |
| *Jup-mouce* | Forward: TGGCAACAGACATACACCTACG |
|  | Reverse: GGTGGTAGTCTTCTTGAGTGTG |
| *Dsp-mouce* | Forward: GGATTCTTCTAGGGAGACTCAGT |
|  | Reverse: TCCACTCGTATTCCGTCTGGG |
| *Dsg1a-mouce* | Forward: ACTGTGTTAAATGTCATCGAGGG |
|  | Reverse: TGCCTGTTCTTGAGTCAACAAC |
| *Dsg1b-mouce* | Forward: GCAGTGGTGGTAATCGTGACC |
|  | Reverse: GGATTTTGCCTACCGGGAGTG |
| *Dsg2-mouce* | Forward: GTGGTCTGCTTGGACTTTGGA |
|  | Reverse: GGAACGGTTTGCCTTCATTTC |
| *Dsg3-mouce* | Forward: TGGCAGTCTGGAAGTCACC |
|  | Reverse: CTGTAGAGGGTCAGGGATGG |
| *Dsc1-mouce* | Forward: GGTCAAGGAATCAAAACACAGC |
|  | Reverse: CCAAGCCGAGGTTGAGTGAAA |
| *Dsc2-mouce* | Forward: ATGGCGGCTGTGGGATCTAT |
|  | Reverse: GCAAGGATCGCAAGGGTCAA |
| *Dsc3-mouce* | Forward: AGTTTGAAAGAGTGTCTCAGCTC |
|  | Reverse: ACAACAGCTCTGGTCGGATAA |
| *Vcl-mouce* | Forward: TGGACGGCAAAGCCATTCC |
|  | Reverse: GCTGGTGGCATATCTCTCTTCAG |
| *Tln1-mouce* | Forward: CCTGCCGCATGATTCGTGA |
|  | Reverse: TCGGAGCATGTAGTAGTCCAAA |
| *Actn1-mouce* | Forward: GACCATTATGATTCCCAGCAGAC |
|  | Reverse: CGGAAGTCCTCTTCGATGTTCTC |
| *Actn4-mouce* | Forward: ATGGTGGACTACCACGCAG |
|  | Reverse: CAGCCTTCCGAAGATGAGAGT |
| *Ptk2-mouce* | Forward: GAGTACGTCCCTATGGTGAAGG |
|  | Reverse: CTCGATCTCTCGATGAGTGCT |
| *Ptk2b-mouce* | Forward: TGAGCCCTTGAGCCGTGTA |
|  | Reverse: AGCTTGAAGTTCTTCCCTGGG |
| *Pxn-mouce* | Forward: CAAACGGCCAGTGTTCTTGTC |
|  | Reverse: TGTGTGGTTTCCAGTTGGGTA |
| *Itga2-mouce* | Forward: TGTCTGGCGTATAATGTTGGC |
|  | Reverse: CTTGTGGGTTCGTAAGCTGCT |
| *Itgb1-mouce* | Forward: ATGCCAAATCTTGCGGAGAAT |
|  | Reverse: TTTGCTGCGATTGGTGACATT |
| *Star-mouce* | Forward: GTGAAGGCTAAGGGATAA |
|  | Reverse: TGGAGCTGGTAAGACAAC |
| *17β-Hsd-mouce* | Forward: CCACCTGTGTTTGGCGTGTA |
|  | Reverse: GAGGTTGAATTGTGGATTAGGCA |
| *Cyp11a1-mouce* | Forward: GGGCAGTTTGGAGTCAGTTTAC |
|  | Reverse: TTTAGGACGATTCGGTCTTTCTT |
| *3β-Hsd-mouce* | Forward: TGGACAAAGTATTCCGACCAGA |
|  | Reverse: GGCACACTTGCTTGAACACAG |
| *Lhcgr-mouce* | Forward: CTGAGGAGATTTGGTTGCTGTA |
|  | Reverse: ATTTGGGTGGACTTTTTTGGGG |
| *β-Actin-mouce* | Forward: CCAGCCTTCCTTCTTGGGTAT |
|  | Reverse: AGGTCTTTACGGATGTCAACG |
| *Gapdh-mouce* | Forward: AGGTCGGTGTGAACGGATTTG |
|  | Reverse: TGTAGACCATGTAGTTGAGGTCA |
| *Vcl-goat* | Forward: GGGTCTTGGAAGCCTAGTGG |
|  | Reverse: AGGCAGACTGGGTTTCTAGC |
| *Tln1-goat* | Forward: GACTGAGGTACAGCAGCGTT |
|  | Reverse: CGGACTCCAAGTGCCTTCAT |
| *Actn1-goat* | Forward: GGGCTTGGTTTCACGCTCTG |
|  | Reverse: GTAAACTGTCACTTCACGGGCA |
| *β-Actin-goat* | Forward: CCTGCGGCATTCACGAAACTAC |
|  | Reverse: ACAGCACCCTGTTGGCGTAGAG |
| *Gapdh-goat* | Forward: GCAAGTTCCACGGCACAG |
|  | Reverse: GGTTCACGCCCATCACAA |
| *VCL-CREBmotif* | Forward: GCCTCTGGAGTGCTGTTGTA |
|  | Reverse: AGGGAAGTGGATCAGGGGTT |
| *TLN1-CREBmotif* | Forward: CTCTGATAATGCCTGTCAAACTTGG |
|  | Reverse: TGCTTCTAACTCCCTCGGACT |
| *INFU-VCL* | Forward: gagctcttacgcgtgctagcCTACCTGCTTCTGTCTCTGAAGTGC |
|  | Reverse: acagtaccggaatgccaagcCAGCCCAGAGAAATCGGCA |
| *INFU-TLN1* | Forward: gagctcttacgcgtgctagcATACTCTCCACAGCCTCTCTGTCAG |
|  | Reverse: acagtaccggaatgccaagcCCGAGTTGGAGAGAACTCCA |
| *Mut-INFU-VCL* | Forward: CAGGATGGaCTCAAACTTTCTTTGTAGTGGAGTATGA |
|  | Reverse: AGTTTGAGtCCATCCTGGAGGGGTAGTCCTTT |
| *Mut-INFU-TLN1* | Forward: AGAGTGACcTCATACACTTGTAATCTAGTCCGAGGG |
|  | Reverse: GTGTATGAgGTCACTCTTGGCTAGTGTCCAAGTT |
